# Supplementary material for: Outcomes of vaccinations against respiratory diseases in patients with end-stage renal disease undergoing hemodialysis: A systematic review and meta-analysis
Source: PLoS One. 2023 Feb 9;18(2):e0281160. doi: 10.1371/journal.pone.0281160 (PMC9910685; doi:10.1371/journal.pone.0281160)
Supplement: S1 Protocol — (PDF) [file pone.0281160.s002.pdf]

NIHR | National Institute for Health Research

PROSPERO
International prospective register of systematic reviews

Vaccinations Against Respiratory Diseases in Patients with End-Stage Renal Disease (ESRD) Undergoing Hemodialysis: A Systematic Review

Metalia Puspitasari, Prenali Dwisthi Sattwika, Dzerlina Syanaiscara Rahari, Jarir Atthobari, Nyoman Kertia, Bambang Purwanto

To enable PROSPERO to focus on COVID-19 submissions, this registration record has undergone basic automated checks for eligibility and is published exactly as submitted. This protocol has been amended since registration with changes to the PICOS criteria, data extraction, quality assessment, or data synthesis methods. Previous versions of the registration may be viewed for comparison. PROSPERO has never provided peer review, and usual checking by the PROSPERO team does not endorse content. Therefore, automatically published records should be treated as any other PROSPERO registration. Further detail is provided **here**.

Citation

Metalia Puspitasari, Prenali Dwisthi Sattwika, Dzerlina Syanaiscara Rahari, Jarir Atthobari, Nyoman Kertia, Bambang Purwanto. Vaccinations Against Respiratory Diseases in Patients with End-Stage Renal Disease (ESRD) Undergoing Hemodialysis: A Systematic Review. PROSPERO 2021 CRD42021255983 Available from: https://www.crd.york.ac.uk/prospero/display_record.php?ID=CRD42021255983

Review question

How is the efficacy and safety of vaccination against respiratory disease given to the end-stage renal disease (ESRD) patient undergoing hemodialysis?

Searches [1 change]

We will conduct a systematic literature search to identify trials that could be considered eligible for inclusion in this review. We will search in the electronic databases PubMed and the Cochrane Library (CENTRAL) until the newest date of publication for interventional [non-randomized or randomized controlled trials (RCTs)], and observational study. Restrictions including non-English language and animal studies.
Our search strategy will include the relevant key terms like “end stage renal disease”, “hemodialysis”, and “vaccine”. We will use the keywords/MeSH terms in the key papers obtained from the PubMed reminder to adapt the searching strategies. In addition, the reference lists of relevant studies will also be searched for further material for inclusion.

Types of study to be included [1 change]

All types of intervention studies (non-randomized intervention study or randomized controlled trials) or observational studies will be considered.

Condition or domain being studied

Patients with end-stage renal disease support their life by hemodialysis to eliminate waste and extra fluid from their bodies. This situation puts the patient in an immunocompromised condition because of the disease's nature. The hemodialysis procedure also puts patients in high-risk infection circumstances because they need to go to the hospital regularly and meet a lot of people. Therefore, these patients are at higher risk of respiratory disease like pneumonia or influenza and their associated outcomes, e.g., death

from respiratory disease. It is therefore important to evaluate the efficacy and safety of strategies like vaccination which could potentially reduce the risk of respiratory disease and its associated outcomes.

Participants/population

Adult ESRD patients undergoing hemodialysis.

Intervention(s), exposure(s) [1 change]

Vaccination against respiratory disease [e.g., influenza vaccine (H3N2, H1N1), pneumococcal vaccine, and Covid-19 vaccine].

Comparator(s)/control [1 change]

Healthy population

Main outcome(s) [1 change]

Primary outcome: antibody seroconversion, antibody seroprotection, respiratory infection
Secondary outcome: safety outcomes (adverse event and serious adverse event)

Measures of effect

We will use the relative risk (RR) of safety outcomes to estimate the effect measures.

Additional outcome(s)

None

Measures of effect

None

Data extraction (selection and coding)

Pairs of reviewers will screen titles and abstract independently to identify studies for inclusion using Covidence software. The eligible or potential eligible studies will be coded as retrieve, which mean that we will retrieve the full text of those studies. Pairs of reviewers will review the full text of retrieved studies to identify the studies that fulfill the eligibility criteria. Pairs of reviewers will use Covidence to extract the characteristic of the study and outcome data, independently. We will resolve any disagreement through discussion and involve the third person if needed. The selection process will be presented as a PRISMA flow chart.

Risk of bias (quality) assessment

The risk of bias of included RCTs will be assessed using the Cochrane risk of bias tool (RoB 2.0). The major domain of bias will be independently assessed including random sequence generation, allocation concealment, blinding, incomplete outcome data, selective outcome reporting and other bias. The risk of bias of included observational studies will be assessed using the Risk of Bias in Non-randomized Studies - of Interventions (ROBINS-I) tool. The major domains of bias will be independently assessed including bias due to confounding, bias in selection of patients into the study, bias due to missing data, bias in measurement of outcomes, bias in selection of the reported results. Risk of bias assessment will be conducted independently by two reviewers. Any inconsistency will be resolved through consensus and a tie-breaker involving a third reviewer will be undertaken, if necessary. These tools will be used in the sensitivity analysis by eliminating studies with high or unclear risk of certain bias.

Strategy for data synthesis

We will use Review Manager 5.4 for analysis the data. The Meta-analysis will be applied when more than one study provides usable data in any single comparison, otherwise we will use narrative analysis. We will include a 95% confidence interval (CI) for all estimates in meta-analysis.

Analysis of subgroups or subsets

The possibility and method for subgroup analysis can be determined after data extraction. Sensitivity analysis will be applied when heterogeneity exists.

Contact details for further information

Metalia Puspitasari
metaliapuspitasari@ugm.ac.id

Organisational affiliation of the review

Division of Nephrology and Hypertension, Department of Internal Medicine, Faculty of Medicine, Public Health, and Nursing, Universitas Gadjah Mada/Dr. Sardjito General Hospital, Yogyakarta, Indonesia

Review team members and their organisational affiliations [1 change]

Mrs Metalia Puspitasari. Division of Nephrology and Hypertension, Department of Internal Medicine, Faculty of Medicine, Public Health, and Nursing, Universitas Gadjah Mada/Dr. Sardjito General Hospital, Yogyakarta, Indonesia
Mrs Prenali Dwisthi Sattwika. Department of Internal Medicine, Faculty of Medicine, Public Health, and Nursing, Universitas Gadjah Mada/Dr. Sardjito General Hospital, Yogyakarta, Indonesia
Ms Dzerlina Syanaiscara Rahari. Faculty of Medicine, Public Health, and Nursing, Universitas Gadjah Mada, Yogyakarta, Indonesia
Mr Jarir Atthobari. Department of Pharmacology and Therapy, Faculty of Medicine, Public Health, and Nursing, Universitas Gadjah Mada, Yogyakarta, Indonesia
Professor Nyoman Kertia. Division of Rheumatology, Department of Internal Medicine, Faculty of Medicine, Public Health, and Nursing, Universitas Gadjah Mada/Dr. Sardjito General Hospital, Yogyakarta, Indonesia
Professor Bambang Purwanto. Division of Nephrology and Hypertension, Department of Internal Medicine, Faculty of Medicine, Universitas Sebelas Maret, Surakarta, Indonesia

Type and method of review

Intervention, Meta-analysis, Methodology, Systematic review

Anticipated or actual start date

01 April 2021

Anticipated completion date

31 December 2021

Funding sources/sponsors

No funding is being received to support the conduct of this review.

Conflicts of interest

Language [1 change]

English

Country

Indonesia

Stage of review

Review Ongoing

Subject index terms status

Subject indexing assigned by CRD

Subject index terms

Humans; Kidney Failure, Chronic; Renal Dialysis; Vaccination

Date of registration in PROSPERO

20 June 2021

Date of first submission

20 May 2021

Stage of review at time of this submission

Stage	Started	Completed
Preliminary searches	Yes	No
Piloting of the study selection process	Yes	No
Formal screening of search results against eligibility criteria	No	No
Data extraction	No	No
Risk of bias (quality) assessment	No	No
Data analysis	No	No

Revision note

We add the words â€œundergoing hemodialysisâ€? on the title to specify the participants of ESRD patients with hemodialysis so the results can be applied to a specific population. We changed the comparator/control group from any population to a healthy population to make it more specific to investigate the natural causes of the decreased immune response, whether it comes from kidney disorders or not (negative control). We add antibody seroprotection to the primary outcome criteria to make it more applicable and accommodate more outcome data of vaccination.

The record owner confirms that the information they have supplied for this submission is accurate and complete and they understand that deliberate provision of inaccurate information or omission of data may be construed as scientific misconduct.

The record owner confirms that they will update the status of the review when it is completed and will add publication details in due course.

Versions

20 June 2021
20 June 2021
04 August 2022

PROSPERO
This information has been provided by the named contact for this review. CRD has accepted this information in good faith and registered the review in PROSPERO. The registrant confirms that the information supplied for this submission is accurate and complete. CRD bears no responsibility or liability for the content of this registration record, any associated files or external websites.
