# Supplementary material for: Outcomes of vaccinations against respiratory diseases in patients with end-stage renal disease undergoing hemodialysis: A systematic review and meta-analysis
Source: PLoS One. 2023 Feb 9;18(2):e0281160. doi: 10.1371/journal.pone.0281160 (PMC9910685; doi:10.1371/journal.pone.0281160)
Supplement: S1 Appendix — (PDF) [file pone.0281160.s003.pdf]

**S2 Appendix. Database Searching Strategy.**

**PubMed Searching Strategy**

1. (end stage renal disease [MeSH Terms]) OR (end stage renal failure[MeSH Terms]) OR (end stage kidney disease[MeSH Terms]) OR (ESRD[Title/Abstract]) OR (end stage renal disease[Title/Abstract]) OR (end stage renal failure[Title/Abstract]) OR (end stage kidney disease[Title/Abstract])
2. (dialysis[MeSH Terms]) OR (renal dialysis[MeSH Terms]) OR (dialys*[Title/Abstract]) OR (renal dialys*[Title/Abstract]) OR (hemodialys*[Title/Abstract]) OR (haemodialys*[Title/Abstract])
3. #1 AND #2
4. (pneumococcal vaccines[MeSH Terms]) OR (influenza vaccines[MeSH Terms]) OR (COVID-19 vaccines[MeSH Terms]) OR (SARS-CoV-2 vaccin*[Title/Abstract]) OR (pneumococcal vaccin*[Title/Abstract]) OR (influenza vaccin*[Title/Abstract]) OR (covid-19 vaccin*[Title/Abstract]) OR (SARS-CoV-2 vaccin*[Title/Abstract])
5. #3 AND #4

**CENTRAL Searching Strategy**

1. (ESRD):ti,ab,kw OR ("end-stage renal disease"):ti,ab,kw OR (end stage renal failure):ti,ab,kw OR (end stage kidney disease):ti,ab,kw OR (dialys*):ti,ab,kw
2. (renal dialys*):ti,ab,kw OR (hemodialys*):ti,ab,kw OR (haemodialys*):ti,ab,kw
3. MeSH descriptor: [Kidney Failure, Chronic] explode all trees
4. MeSH descriptor: [Dialysis] explode all trees
5. MeSH descriptor: [Renal Dialysis] explode all trees
6. #1 OR #3
7. #2 OR #4 OR #5
8. #6 AND #7
9. MeSH descriptor: [Pneumococcal Vaccines] explode all trees
10. MeSH descriptor: [Influenza Vaccines] explode all trees
11. MeSH descriptor: [COVID-19 Vaccines] explode all trees
12. (pneumococcal vaccin*):ti,ab,kw
13. (influenza vaccin*):ti,ab,kw
14. (covid-19 vaccin*):ti,ab,kw
15. (sars-cov-2 vaccin*):ti,ab,kw
16. #9 OR #10 OR #11 OR #12 OR #13 OR #14 OR #15
17. #8 AND #16

**Scopus Searching Strategy**

1. ( TITLE-ABS-KEY ( end AND stage AND renal AND disease ) OR TITLE-ABS-KE Y ( end AND stage AND renal AND failure ) OR TITLE-ABS-KEY ( end AND stage A

ND kidney AND disease ) OR TITLE-ABS-KEY ( end AND stage AND kidney AND failure ) OR TITLE-ABS-KEY ( esrd )
2. TITLE-ABS-KEY ( renal AND dialys* ) OR TITLE-ABS-KEY ( hemodialys* ) OR TITLE-ABS-KEY ( dialys* ) OR TITLE-ABS-KEY ( haemodialys* )
3. #1 AND #2
4. TITLE-ABS-KEY ( pneumococcal AND vaccin* ) OR TITLE-ABS-KEY ( influenza AND vaccin* ) OR TITLE-ABS-KEY ( covid-19 AND vaccin* ) OR TITLE-ABS-KEY ( sars-cov-2 AND vaccin* )
5. #3 AND #4

**ScienceDirect Searching Strategy**
1. "end stage renal disease" OR "end stage renal failure" OR "end stage kidney disease" OR ESRD
2. dialysis OR hemodialysis OR haemodialysis
3. #1 AND #2
4. "pneumococcal vaccine" OR "influenza vaccine" OR "COVID-19 vaccine" OR "SARS-CoV-2 vaccine"
5. #3 AND #4

**ProQuest Searching Strategy**
1. summary(ESRD) OR summary(end stage renal disease) OR summary(end stage renal failure) OR summary(end stage kidney disease)
2. summary(dialys*) OR summary(hemodialys*) OR summary(haemodialys*)
3. #1 AND #2
4. summary(pneumococcal vaccin*) OR summary(influenza vaccin*) OR summary(covid-19 vaccin*) OR summary(sars-cov-2 vaccin*)
5. #3 AND #4

**Google Scholar Searching Strategy**
1. "end stage renal disease" OR "end stage renal failure" OR "end stage kidney disease"
2. dialysis OR hemodialysis OR haemodialysis
3. #1 AND #2
4. "pneumococcal vaccines" OR "influenza vaccines" OR "covid-19 vaccines" OR "SARS-CoV-2 vaccines"
5. #3 AND #4
