# Supplementary material for: Outcomes of vaccinations against respiratory diseases in patients with end-stage renal disease undergoing hemodialysis: A systematic review and meta-analysis
Source: PLoS One. 2023 Feb 9;18(2):e0281160. doi: 10.1371/journal.pone.0281160 (PMC9910685; doi:10.1371/journal.pone.0281160)

S4 Appendix. Funnel plots of studies included in the meta-analyses.

(a) Funnel plots of studies assessing seroconversion rate after H1N1 vaccination in ESRD patients undergoing hemodialysis.

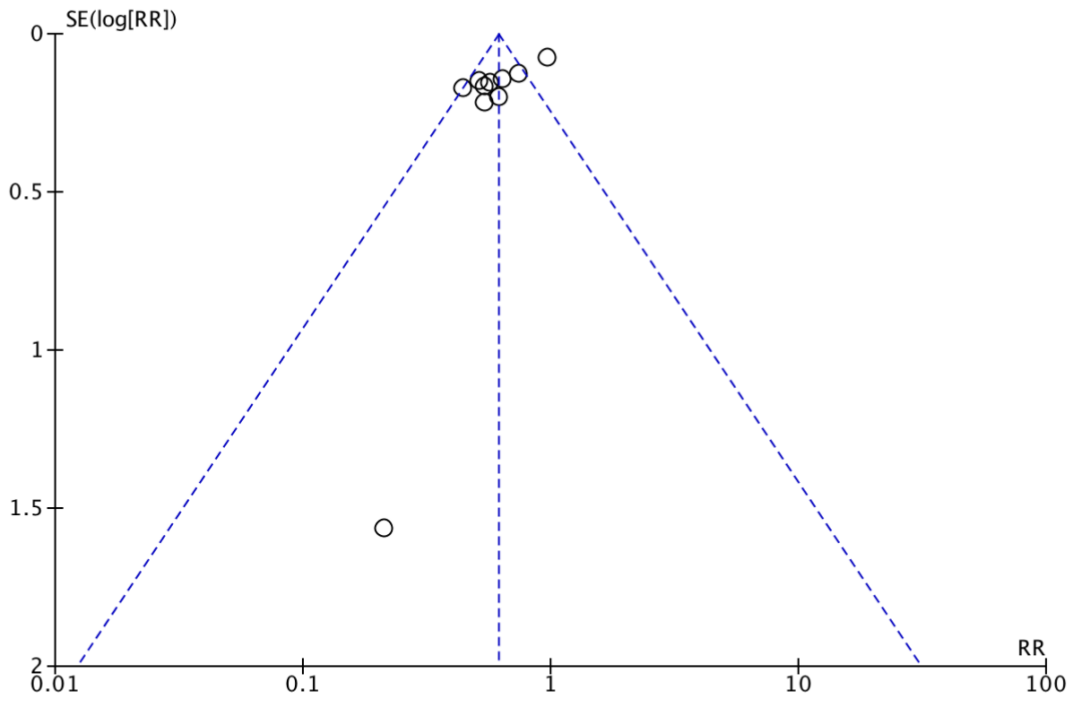

(b) Funnel plots of studies assessing seroprotection rate after H1N1 vaccination in ESRD patients undergoing hemodialysis.

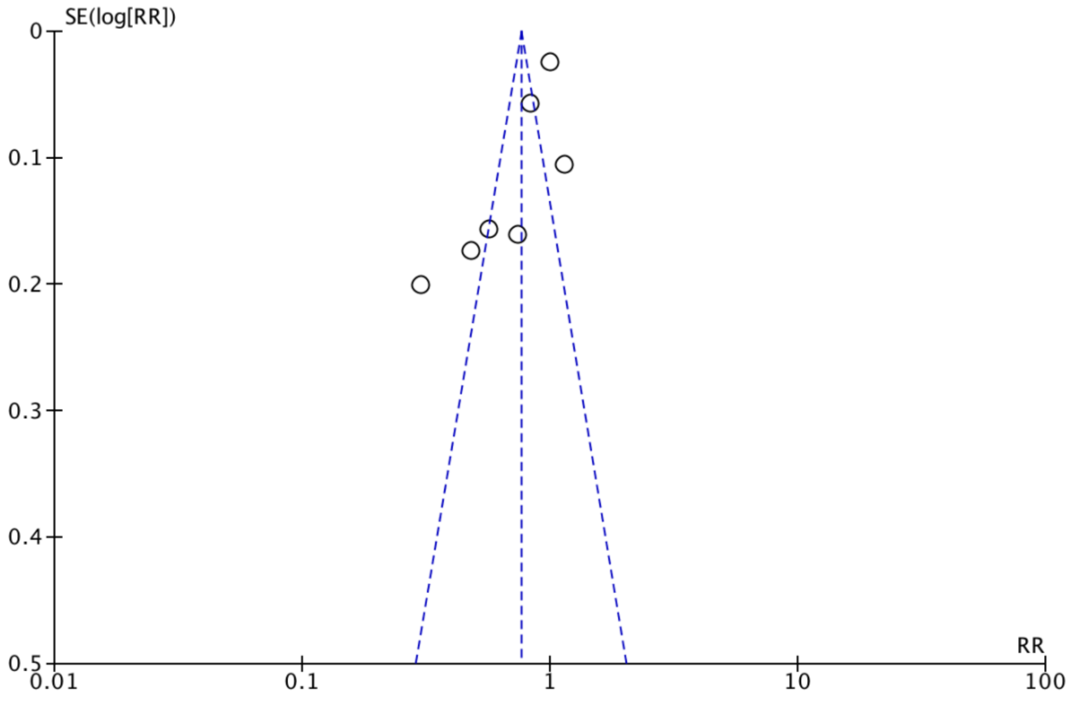

(c) Funnel plots of studies assessing seroconversion rate after H3N2 vaccination in ESRD patients undergoing hemodialysis.

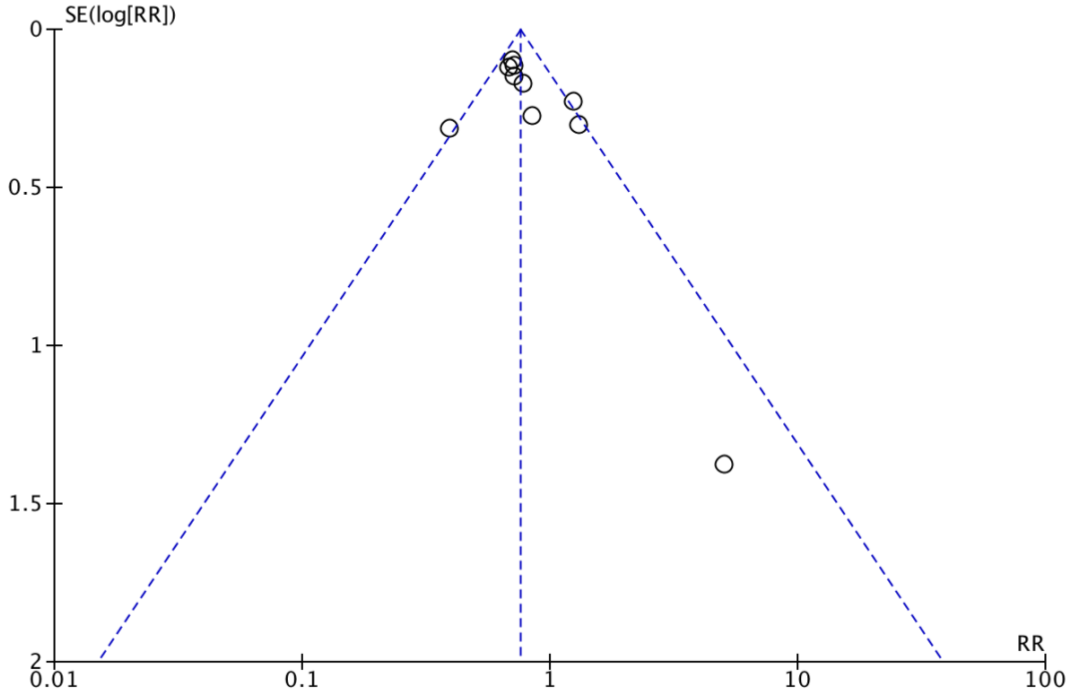

(d) Funnel plots of studies assessing seroprotection rate after H3N2 vaccination in ESRD patients undergoing hemodialysis.

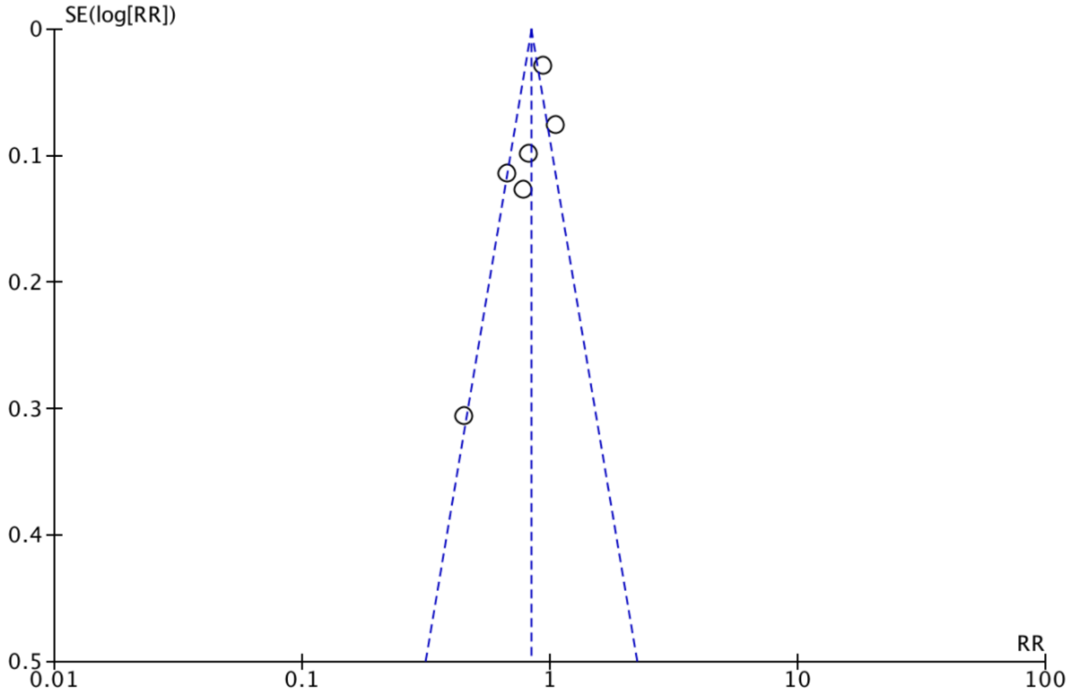

(e) Funnel plots of studies assessing adverse events after COVID-19 vaccination in ESRD patients undergoing hemodialysis.

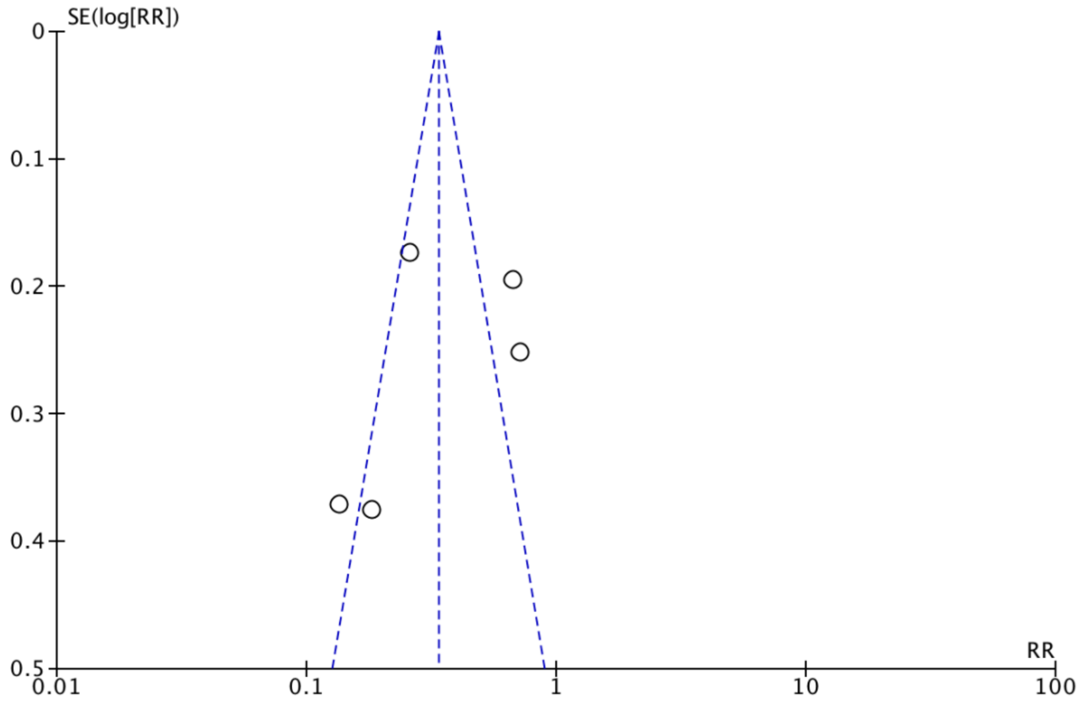

Supplement: S2 Appendix — (PDF) [file pone.0281160.s004.pdf]
