# Supplementary material for: Outcomes of vaccinations against respiratory diseases in patients with end-stage renal disease undergoing hemodialysis: A systematic review and meta-analysis
Source: PLoS One. 2023 Feb 9;18(2):e0281160. doi: 10.1371/journal.pone.0281160 (PMC9910685; doi:10.1371/journal.pone.0281160)
Supplement: S1 Table — (PDF) [file pone.0281160.s005.pdf]

S4 Table. Grading of Recommendations, Assessment, Development, and Evaluation (GRADE) criteria for studies included in the meta-analyses.

(A) Meta-analysis of studies assessing seroconversion rate after H1N1 vaccination in ESRD patients undergoing hemodialysis

Number of participants	Starting level of evidence	Quality assessment					Reasons to increase level of evidence (Large magnitude of effect; Dose-response gradient; Potential confounding)	Overall quality of evidence
		Risk of bias	Inconsistency	Indirectness	Imprecision	Publication bias		
1191	Low	Not serious	Not serious	Not serious	Not serious	Serious	N/A	Very low

(B) Meta-analysis of studies assessing seroprotection rate after H1N1 vaccination in ESRD patients undergoing hemodialysis

Number of participants	Starting level of evidence	Quality assessment					Reasons to increase level of evidence (Large magnitude of effect; Dose-response gradient; Potential confounding)	Overall quality of evidence
		Risk of bias	Inconsistency	Indirectness	Imprecision	Publication bias		
1001	Low	Serious	Serious	Not serious	Not serious	Serious	N/A	Very low

(C) Meta-analysis of studies assessing seroconversion rate after H3N2 vaccination in ESRD patients undergoing hemodialysis

Number of participants	Starting level of evidence	Quality assessment					Reasons to increase level of evidence (Large magnitude of effect; Dose-response gradient; Potential confounding)	Overall quality of evidence
		Risk of bias	Inconsistency	Indirectness	Imprecision	Publication bias		
1012	Low	Serious	Serious	Not serious	Serious	Serious	N/A	Very low

(D) Meta-analysis of studies assessing seroprotection rate after H3N2 vaccination in ESRD patients undergoing hemodialysis

Number of participants	Starting level of evidence	Quality assessment					Reasons to increase level of evidence (Large magnitude of effect; Dose-response gradient; Potential confounding)	Overall quality of evidence
		Risk of bias	Inconsistency	Indirectness	Imprecision	Publication bias		
691	Low	Not serious	Serious	Not serious	Not serious	Serious	N/A	Very low

(E) Meta-analysis of studies assessing adverse events rates after COVID-19 vaccination in ESRD patients undergoing hemodialysis

Number of participants	Starting level of evidence	Quality assessment					Reasons to increase level of evidence (Large magnitude of effect; Dose-response gradient; Potential confounding)	Overall quality of evidence
		Risk of bias	Inconsistency	Indirectness	Imprecision	Publication bias		
677	Low	Not serious	Serious	Not serious	Not serious	Not serious	N/A	Very low
